# Utility of the NavX® Electroanatomic Mapping System for Permanent Pacemaker Implantation in a Pregnant Patient with Chagas Disease

**DOI:** 10.1016/s0972-6292(16)30586-1

**Published:** 2013-01-01

**Authors:** Alejandro Velasco, Victor Manuel Velasco, Fernando Rosas, Cihan Cevik, Carlos A Morillo

**Affiliations:** 1 Internal Medicine Department. Texas Tech University Health Sciences Center, Lubbock, Texas. USA; 2 Electrophysiology and Cardiac Stimulation Service. Fundacion Clinica Shaio, Bogota. Colombia; 3 Cardiology Department, Texas Heart Institute at St.Luke's Episcopal Hospital, Baylor College of Medicine, Houston, Texas. USA; 4 Arrhythmia Services, Hamilton Health Sciences. Mc Master University, Hamilton, Ontario. Canada

**Keywords:** Chagas disease, Heart Block, Pregnancy, Electroanatomic Navigation, Permanent Cardiac Pacemaker, Non-fluoroscopic imaging

## Abstract

Chagas disease is a highly prevalent zoonosis in Mexico, Central, and South America. Early cardiac involvement is one of the most serious complications of this disease, and conduction disturbances may occur at an early age. We describe a young pregnant woman with Chagas disease and a high degree atrioventricular block, who required implantation of a permanent dual chamber pacemaker. Using an electroanatomic navigation EnSite NavX® system the pacemaker was successfully implanted with minimal fluoroscopic exposure. This case demonstrates the safety and feasibility of using an electroanatomic navigation system to guide permanent pacemaker implantation minimizing x-ray exposure in pregnant patients.

## Introduction

Chagas Disease is a highly prevalent endemic zoonosis in most of Mexico, Central, and South America. It is caused by a parasite transmitted to humans by insects, blood transfusions, oral secretions, or across the placenta. Cardiac involvement is one of the most serious complications of chronic infection by Chagas disease, leading to a chronic dilated cardiomyopathy, which can present with multiple conduction disorders including atrioventricular (AV) blocks. Cardiomyopathy develops approximately 20 years after acute infection, putting fertile women at risk of having cardiac rhythm disturbances during pregnancy if infected at an early age [1]. There are no established recommendations to manage these types of patients when they present with high degree AV block. The new imaging techniques for ablation of cardiac arrhythmias reduce the exposure to X-rays, and they may be useful during pacemaker implantation in pregnant patients. We report a young woman with Chagas cardiomyopathy in whom a double chamber pacemaker using an electroanatomic navigation system was performed to avoid fluoroscopy and protect the fetus from radiation exposure.

## Case

**Figure 1:**
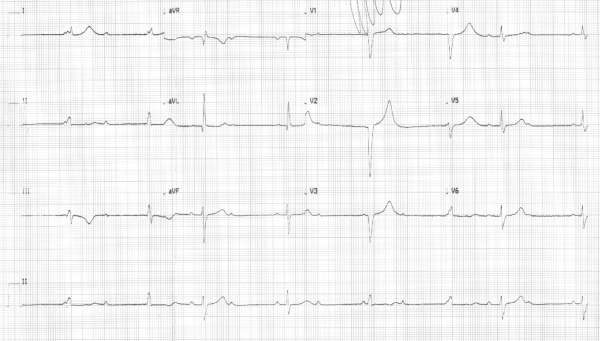
Twelve-lead electrocardiogram at presentation shows a third degree AV block

A 33 -year-old pregnant woman was referred with the diagnosis of bradycardia. She presented with a 2 months history of fatigue; during that time she was found to have positive antibodies for Chagas disease. At the time of admission she had a 31 weeks gestation. Her past medical history was only remarkable for a previous cesarean section. At physical exam a resting heart rate of 33 bpm was noted, and her ECG showed a third degree AV block (Figure 1). By Ultrasound the fetus showed intrauterine growth delay.

**Figure 2:**
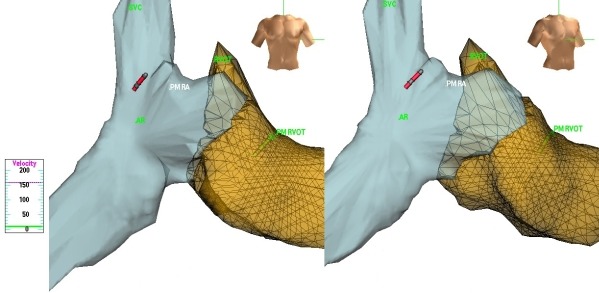
Anterior and right anterior oblique views of the patient's heart showing the position of both pacemaker leads. SVC: Superior Vena Cava AR: Right Atria PM RA: Right Atrial Lead RVOT: Right ventricular outflow tract PM RVOT: Right ventricle lead

We decided to implant a dual chamber permanent pacemaker. To minimize the X-ray exposure, we used an electroanatomic navigation system ENSITE NavX® System (Endocardial Solutions Inc., USA) during pacemaker implantation. A written informed consent was obtained from the patient. Three pairs of adhesive electrodes were placed on the patient chest to create the electrical field needed to create the navigation field. Under local anesthesia, two left subclavian punctures were performed, and a quadripolar steerable catheter was advanced through the sheath (IBI-83405, 7 Fr., 2.3 mm. Irvine Biomedical Inc., St. Jude Medical, USA). Contours of the superior vena cava, right atrium, tricuspid annulus, right ventricular apex and right ventricular outflow tract were created with the quadripolar catheter. The quadripolar catheter was then withdrawn, and an 8 Fr. replaced the 7 Fr. introducer. To visualize the atrial and ventricular leads, two cables were used with alligator clips attached to the pacemaker leads and a 2 mm pin end attached to the Ensite NavX® polygraph box. By being attached to the polygraph box, the leads were visualized as catheters within the electroanatomic contour. An active fixation ventricular lead (Tendril ST 58 cm, St. Jude Medical, USA) was advanced using the electroanatomic images as a reference and was placed in the right ventricular outflow tract. An R wave of 4.8 mV, a threshold of 0.5 V, and an impedance of 357 ohms in bipolar mode were documented after implantation. A second introducer was advanced, and an active fixation atrial lead (Tendril ST 52cm, St Jude Medical, USA) was positioned guided by the electroanatomic map. A P wave of 6.5 mV, a threshold of 1.2 V, and an impedance of 481 ohms in bipolar mode were obtained. Three-dimensional map documenting the final position of both leads is shown (Figure 2). The position of the electrodes was verified by the P wave and QRS morphology during pacing. Fluoroscopy for 5 seconds was performed to confirm active fixation deployment and lead positioning. Safety precautions for the baby were taken with a lead apron placed over the mother's abdominal area to minimize radiation exposure. Electrodes were connected to a Zaphyr pacemaker (St. Jude Medical) in the DDDR mode.

**Figure 3:**
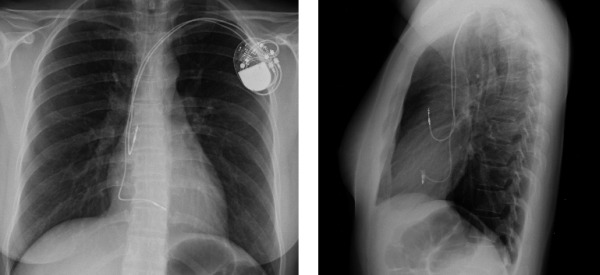
Chest x-ray demonstrates adequate position of the atrial and ventricular leads after delivery of pregnancy

Six months after the procedure the pacemaker was functioning correctly (atrial pacing < 1%, ventricular pacing > 99%, impedance A: 378 ohms V: 384 ohms, atrial threshold <0.25V and ventricular threshold 0.75 V). The baby was uneventfully delivered at term by a cesarean section. The position of the leads was confirmed after delivery with a chest x-ray (Figure 3). The newborn had a positive serology for Chagas disease and was treated with Benznidazol 5 mg/kg/d for 30 days.

## Discussion

A permanent pacemaker is the treatment of choice in symptomatic pregnant patients with high degree AV block [2]. However, there is an unavoidable concern about harm using x-rays during pregnancy. Ultrasound guidance can be used for placing transvenous pacemaker leads in pregnant women. However the procedure may take significantly more time if transesophageal echocardiography is used and there is a high risk for aspiration due to delayed gastric emptying in pregnancy. Transthoracic echocardiography can be useful with the aid of electrocardiographic signals; however, it is very difficult to visualize atrial leads with this technique [3]. The electroanatomic navigation system NavX® has emerged as an important tool in the management of complex arrhythmias and may decrease the overall exposure to fluoroscopy. This navigation tool is based on the creation of an electric voltage gradient between electrodes placed on the chest of the patient. This creates a three dimensional map that visualizes the position of catheters inserted to the patients, and with fine movements it can reproduce the morphology of the cardiac chambers. Small case series have described the use of NavX® for single lead permanent pacemakers with an acceptable accuracy rate and without a significant increase in procedural or fluoroscopic time [4,5]. To the best of our knowledge this is the first case of a dual chamber permanent pacemaker implantation using the NavX® system in a pregnant patient with Chagas cardiomyopathy.

This case of a pregnant woman with complete heart block secondary to Chagas cardiomyopathy demonstrates the feasibility of using the NavX® mapping system to guide the insertion of a permanent dual chamber pacemaker and reduce the risk of X-Ray exposure to the fetus. This approach seems safe, feasible and can be considered as first line option in pregnant patients when permanent pacemaker implantation is needed.
